# MicroRNA-142-5p contributes to Hashimoto’s thyroiditis by targeting CLDN1

**DOI:** 10.1186/s12967-016-0917-6

**Published:** 2016-06-08

**Authors:** Jin Zhu, Yuehua Zhang, Weichen Zhang, Wei Zhang, Linni Fan, Lu Wang, Yixiong Liu, Shasha Liu, Ying Guo, Yingmei Wang, Jun Yi, Qingguo Yan, Zhe Wang, Gaosheng Huang

**Affiliations:** State Key Laboratory of Cancer Biology and Department of Pathology, Xijing Hospital, Fourth Military Medical University, Changle West Road #169, Xi’an, 710032 People’s Republic of China; Department of Clinical Laboratory, Lintong Sanatorium, Lanzhou Military Command, Xi’an, 710600 People’s Republic of China; Department of Pathology, Foshan First People’s Hospital, Foshan, 528000 People’s Republic of China; The Helmholtz Sino-German Laboratory for Cancer Research, Department of Pathology, Tangdu Hospital, Fourth Military Medical University, Xi’an, 710038 People’s Republic of China; Department of Neurology, Tangdu Hospital, Fourth Military Medical University, Xi’an, 710038 People’s Republic of China; Department of Vascular and Endocrine Surgery, Xijing Hospital, Fourth Military Medical University, Xi’an, 710032 People’s Republic of China

**Keywords:** Autoimmune diseases, Hashimoto’s thyroiditis, miRNA, miR-142-5p, CLDN1

## Abstract

**Background:**

MicroRNAs have the potential as diagnostic biomarkers and therapeutic targets in autoimmune diseases. However, very limited studies have evaluated the expression of microRNA profile in thyroid gland related to Hashimoto’s thyroiditis (HT).

**Methods:**

MicroRNA microarray expression profiling was performed and validated by quantitative RT-PCR. The expression pattern of miR-142-5p was detected using locked nucleic acid-in situ hybridization. The target gene was predicted and validated using miRNA targets prediction database, gene expression analysis, quantitative RT-PCR, western blot, and luciferase assay. The potential mechanisms of miR-142-5p were studied using immunohistochemistry, immunofluorescence, and quantitative assay of thyrocyte permeability.

**Results:**

Thirty-nine microRNAs were differentially expressed in HT (Fold change ≥2, P < 0.05) and miR-142-5p, miR-142-3p, and miR-146a were only high expression in HT thyroid gland (P < 0.001). miR-142-5p, which was expressed at high levels in injured follicular epithelial cells, was also detected in HT patient serum and positively correlated with thyroglobulin antibody (r ≥ 0.6, P < 0.05). Furthermore, luciferase assay demonstrated CLDN1 was the direct target gene of miR-142-5p (P < 0.05), and Immunohistochemical staining showed a reverse expression patterns with miR-142-5p and CLDN1. Overexpression of miR-142-5p in thyrocytes resulted in reducing of the expression of claudin-1 both in mRNA and protein level (P = 0.032 and P = 0.009 respectively) and increasing the permeability of thyrocytes monolayer (P < 0.01).

**Conclusions:**

Our findings indicate a previously unrecognized mechanism that miR-142-5p, targeting CLDN1, plays an important role in HT pathogenesis.

**Electronic supplementary material:**

The online version of this article (doi:10.1186/s12967-016-0917-6) contains supplementary material, which is available to authorized users.

## Background

Hashimoto’s thyroiditis (HT) was first described by the Japanese physician Hakaru Hashimoto in 1912 [1]. Thereafter it was recognized as the first and most common organ-specific autoimmune disease in the world, and its prevalence has increased in recent years [2]. In medical practice, HT is the most common cause of primary hypothyroidism and is related to the development of both thyroid carcinoma and lymphoma. It is important therefore to understand the pathogenesis of HT. Although the exact etiology of the immune response was unknown for more than 100 years, characteristic lesions, including a large amount of lymphoid tissue infiltration and thyroid follicular cell injury (Hürthle cell formation), indicated that the thyroid gland was attacked by various cell- and antibody-mediated autoimmune responses. Recently, there has been great progress in the identification of several major genes and environmental factors which contribute to the etiology of HT [3]. However, little is known about the roles of functional noncoding sequences related to HT, particularly those of microRNAs (miRNAs).

miRNAs are recently discovered, small, noncoding RNAs of approx. 22 nucleotides that emerged as a new class of modulators of gene expression at the posttranscriptional level. The function of miRNAs is to bind to the 3ʹ untranslated regions (UTRs) of target mRNAs and either prevent their translation or cause their degradation. Accumulating data suggest that miRNAs are differentially expressed in autoimmune diseases and miRNA regulation may impact their development or prevention [4], including rheumatoid arthritis, systemic lupus erythematosus, primary Sjögren’s syndrome, multiple sclerosis, ulcerative colitis, and inflammatory bowel disease. However, the research on HT-related miRNAs is not only very limited, but also not direct study on thyroid gland. Until recently, except for few studies finding dysregulated expression of miRNA in the peripheral blood mononuclear cells and serum of HT patients [5–8], only two pilot study reported that three miRNAs had dysregulated expression in HT using 10 samples of fine-needle aspiration biopsies [9] and 21 laser capture microdissection samples [10]. We are afraid that maybe the results were not representative to some degree quite probably because of the uneven distribution of the characteristic lesions obtained from fine-needle aspiration biopsies and the limitations due to their limited candidate miRNAs and the small sample size.

As the direct target organs of HT, the HT-associated miRNAs expression profile of the lesion in thyroid tissue remains totally unclear, and theoretically, understanding the expression profile and the disease target organ-specific miRNA is crucial to the function and mechanism research of HT. Therefore, we investigated the levels of miRNAs in the HT patients and healthy controls using more than 70 paraffin-embedded tissues obtained from surgical operations.

## Methods

### Tissue samples

We retrospectively studied consecutive 142 formalin-fixed paraffin-embedded (FFPE) thyroid tissues samples between 2010 and 2013 from the Department of Pathology, Xijing hospital. Among the 142 FFPE thyroid tissues samples, there were 71 cases of HT, 20 cases of papillary thyroid carcinoma (PTC), 30 cases of nodular goiter, and 21 cases of normal thyroid tissue obtained from adjacent adenoma normal tissues of patients with thyroid adenoma. Among the 71 HT tissues samples, 42 were primary HT, 14 HT concomitant PTC, and 15 HT concomitant nodular goiter. Patient characteristics in the different cohorts are summarized in Additional file 1: Table S1. All thyroid tissue samples were reviewed by at least two experienced pathologists to confirm the diagnosis of HT. The pathological diagnostic criteria of HT depended on characteristic pathological appearance. The main feature is infiltration with lymphocytes, organized in lymphoid follicles that often show prominent germinal centers. Meanwhile it was accompanied with the transformation of normal thyrocytes into Hürthle cells in some areas, destruction and atrophy of thyrocytes in other areas, and interstitial fibrosis [11, 12].

Among the 71 HT tissues samples, only 29 cases had the complete clinical records for the correlation analysis between the expression of miRNAs in thyroid tissues and the expression of serum markers. Due to the different patients chose different serological detection method of serum markers and the two methods had their own reference standard, we divided clinical data into two groups, one for chemiluminescence detection (n = 17) and the other for radioimmune (n = 12).

Serum samples for miRNA analysis were obtained from five stored patients serum of HT without any medication (two of them were HT with papillary thyroid carcinoma) and six healthy donors.

Approvals for the study were obtained from the Ethics Committees at Fourth Military Medical University.

### RNA isolation and microarray analysis

Total RNA for miRNA detection was isolated using RecoverAll™ Kit (AM1975; Ambion, USA) from FFPE samples and mirVana™ PARIS™ kit (AM1556; Ambion) for cells or body fluid according to the manufacturer’s protocol. The Human miRNA Microarray (Sanger miRBase release 12.0; Agilent, USA) was used to generate the miRNA expression profile in the thyroid tissues. The results were analyzed using the Student’s t test to identify significant differences between HT and normal thyroid (*P* < 0.05).

Total RNA for mRNA detection was extracted from cells with RNAiso Plus (TaKaRa, Japan) according to the manufacturer’s protocol. The PrimeView™ Human Gene Expression Array (Affymetrix, USA) was used to identify the different gene expression in thyrocytes upregulated with miR-142-5p.

### Validating microarray results by quantitative RT-PCR analysis (qRT-PCR)

cDNA was synthesized using a One Step PrimeScript^®^ miRNA cDNA Synthesis Kit for miRNA and a High-Capacity cDNA Reverse Transcription Kit for mRNA (TaKaRa) according to the manufacturer’s instructions. The products of PCR amplification were detected using SYBR Premix Ex TaqTM^II^ (TaKaRa). The reaction conditions were applied according to the manufacturer’s instructions, of which the annealing temperature used was 60 °C for miRNA and 58 °C for mRNA. Expression of each miRNA or mRNA was quantified by measuring cycle threshold (Ct) values and RNU6B or GAPDH was used as internal control to calculate the relative expression level of differentially expressed miRNAs or mRNAs by the 2^−△△Ct^ method [13]. All samples were completed in triplicate.

### Locked nucleic acid-in situ hybridization (LNA-ISH)

LNA-ISH was performed according to the manufacturer’s instructions and relevant LNA-ISH literature [14, 15]. The 4 µm thick sections of FFPE tissues adhered to glass slides were de-waxed and rehydrated into phosphate buffered saline (PBS). They were then digested with 15 μg/mL proteinase K (Exiqon, Denmark) at 37 °C for 10 min, and rinsed for 3 × 5 min in PBS. Slides were then immersed in 3 % H_2_O_2_ for 30 min at room temperature, then rinsed and dehydrated by stages into ethanol and air dried. LNA-modified probes were obtained from Exiqon, Singly labelled with FITC at the 5ʹ termini or doubly labelled with digoxigenin at the 5ʹ and 3ʹ termini. They were then diluted to 40 nM in 1 × Exiqon hybridization buffer. The probes were denatured at 80 °C for 4 min, and then chilled on ice for 15 min. A 25 μl probe was added to cover the tissue area, then covered with a glass coverslip, and sealed with rubber cement. The slides were then hybridized on a hybridizer for 3 h at 55 °C. Coverslips were removed, and the slides were washed in 5 × SSC for 5 min, then 2 × 5 min in 1 × SSC and 2 × 5 min in 0.2 × SSC, all at hybridization temperature. This was followed by 2 × 5 min washes at room temperature in 0.2 × SSC and a final rinse in 1 × PBS. Sections were then incubated with anti-digoxigenin-peroxidase Fab fragments (Roche, Germany), diluted to 1:150 in blocking buffer (2 % sheep serum, 2 % BSA in 1 × PBS) for 1 h at room temperature. Excess antibody was washed away in TBST buffer (0.1 M Tris–HCl, pH 7.4, 0.15 M NaCl, 0.05 % Tween 20) for 3 × 5 min. For amplification of antibody signals, FITC-conjugated phenol (fluorescyl-tyramide, K1497; DAKO, Denmark) was applied to the slides for 15 min at room temperature, followed by three washes in TBST. Finally, an anti-FITC antibody conjugated to horseradish peroxidase (K1497; DAKO) was added to the slides for 16 min at room temperature, followed by three washes in TBST. The reaction products were visualized using DAB. An equivalent LNA probe in which the miR-142-5p oligonucleotide sequence had been scrambled was used as the negative control.

### Isolation and culture of human thyrocytes

Human tissue was obtained from patients operated on for thyroid adenoma. Isolation and culture of human thyrocytes were performed according to a standard method, as described previously [16], minced adenoma-adjacent normal thyroid tissue was digested with 0.25 mg/mL collagenase in the presence of 0.1 mg/mL trypsin inhibitor type I-S and 2 mg/mL DNase I (Sigma, USA). To remove connective tissue, the cell suspension was filtered through nylon filters with diminishing pore size. Blood cells and thyroid-infiltrating lymphocytes were to a major extent excluded by repeated washings and centrifugations at 600 rpm for 5 min of the follicle preparation. The isolated segments of ruptured thyroid follicles were either frozen in fetal calf serum (Gibco, USA) containing 10 % DMSO (Sigma) and stored at −80 °C before use, or seeded directly onto a plastic support. The cells were cultured in 5 % CO_2_ at 37 °C in DMEM/F12 medium supplemented with 10 % fetal calf serum, penicillin (200 U/mL), and streptomycin (200 U/mL; Gibco), in the presence or absence of bovine TSH, 1 mU/mL; Sigma).

### Generation of lentiviral vectors and miRNA transfer

Lentiviral particles carrying the hsa-mir-142 precursor and its control were purchased from GeneChem (GeneChem Co, Shanghai, China). Lentiviral transduction was carried out according to the manufacturer’s protocols. Briefly, thyrocytes cells were infected with LV-has-mir-142 or LV-GFP in the presence of 4 µg/mL polybrene (Sigma) at a multiplicity of infection of 12 h. Thereafter cells were changed fresh medium and cultured for 72 h. The infection efficiency was evaluated by the expression of GFP in cells observed through fluorescence microscopy, and the expression of mature miR-142-5p or miR-142-3p was confirmed using qRT-PCR.

### Western blot analysis

Thyrocytes grown in 60 mm dishes were infected with LV-mir-142 or LV-GFP for 72 h and lysed with cell disruption buffer (AM1556; Ambion) for 30 min on ice. Protein was quantitated using a BCA protein assay (Pierce, Thermo). Protein aliquots (50 µg per lane) were separated using 12 % SDS/PAGE and transferred onto a PVDF membrane (Millipore, USA). Membranes were blocked in Tris-buffered saline Tween containing 5 % non-fat dry milk and blotted with primary polyclonal antibodies CLDN1 (1:500 dilution; Abcam, UK) or β-actin (1:3000; Santa Cruz, CA, USA) overnight. After washing, membranes were incubated at 37 °C with secondary peroxidase-linked goat anti-rabbit or anti-mouse IgG (1:5000 dilution; Santa Cruz) for 1 h. Protein bands were visualized using an enhanced chemiluminescence kit (Bestbio Co., Shanghai, China) and autoradiography. The staining intensity was quantified using the Gel-Pro Analyzer 4.5 software for calculations.

### Immunohistochemistry and immunofluorescence

Paraffin-embedded sections of 4 µm thick were deparaffinised and treated with heat-mediated antigen retrieval with citrate buffer pH6. Then, 3 % hydrogen peroxide was added to block endogenous peroxidase activity at room temperature for 30 min, followed by incubation in 5 % BSA (Sigma) in PBS at room temperature for 20 min to block the nonspecific antibody-binding sites. Sections were then incubated overnight at 4 °C with the rabbit polyclonal antibody against human CLDN1 proteins (1:200 dilution; Abcam). Later on, a standard rapid EnVision technique (Dako) was used to detect the protein conjugates and develop the colour. Finally, the sections were visualized after counterstaining with hematoxylin. Serial sections were run in parallel with the primary antibody replaced by PBS as controls.

Human thyrocytes cultured on glass slides were fixed in 100 % ethanol for 10 min followed by washing with PBS and pre-incubation with blocking buffer containing 1 % BSA, 10 % normal goat serum in 0.1 % PBS-Tween for 1 h. The cells were then incubated with rabbit primary antibodies against CLDN1 (1:40 dilution; Abcam) at 4 °C overnight followed by incubation with anti-rabbit secondary antibody conjugated to Cy3 (1:400 dilution; Millipore) for 1 h the next day. Serial sections were run in parallel with the primary antibody replaced by PBS as controls.

### Luciferase assay

Plasmids containing wild-type Luc-CLDN1 and mutant Luc-CLDN1 3ʹ-UTR were specifically synthesized (GeneChem Co.). The Luc-mut vector, in which the seven nucleotides complementary to the miR-142-5p seed-region were mutated by site-directed mutagenesis, was constructed as a mutant control. These plasmids contain firefly luciferase and Renilla luciferase that functions as a tracking gene. Luciferase activity assays were performed following manufacturer’s protocols. Briefly, HEK293T cells were plated in a 24-well plate at a density of 1 × 10^4^/well. After overnight incubation, cells were cotransfected with 4 µg of firefly luciferase reporter vector containing the wild-type or mutant oligonucleotides, 50 nM miR-142-5p mimics, or negative mimics control by using lipofectamine 2000 (Invitrogen, USA). Luciferase activities were measured 48 h after transfection using the Dual Luciferase Reporter Assay System on a luminometer (GloMax TM 20/20; Promega, USA). Relative luciferase activity was normalized to Renilla luciferase activity for each transfected well. The experiments were performed in triplicate in three independent experiments.

### Quantitative assay of thyrocyte permeability

Thyrocytes at 100,000 cells/insert were seeded onto the collagen-coated insert of an in vitro Vascular Permeability Assay kit (ECM644; Millipore) and grown to confluence. After overnight incubation, the thyrocytes were infected with LV-mir-142 or LV-GFP in the presence of 4 µg/ml polybrene for 12 h and then the culture medium was changed. After lentiviral transduction for 72 h, FITC dextran (1:40 dilution) was added to the medium and allowed to permeate through the cell monolayer. The extent of permeability after 20 min was determined using a fluorescent plate reader (Tecan, USA) to measure the FITC content remaining on the plate. After testing, the thyrocyte monolayer was stained for brightfield imaging of monolayer integrity. This procedure was repeated more than three times.

### Statistical analysis

Data analyses were performed using SPSS software Version 17.0 (SPSS Inc., USA). Student’s t tests for two groups or one-way ANOVA were performed to evaluate the statistical significance of clustered miRNAs in each group as well as in the functional assays. Spearman and Pearson correlation tests were used to analyze the relationship between clinical parameters and miRNAs. In cellular studies, values were expressed as mean ± SD. Comparisons between groups were assessed using one-way ANOVA. All experiments were done in triplicate and all tests were two-sided. P < 0.05 were considered statistically significant.

## Results

### miRNA expression profile of HT vs. normal thyroid

**Fig. 1:**
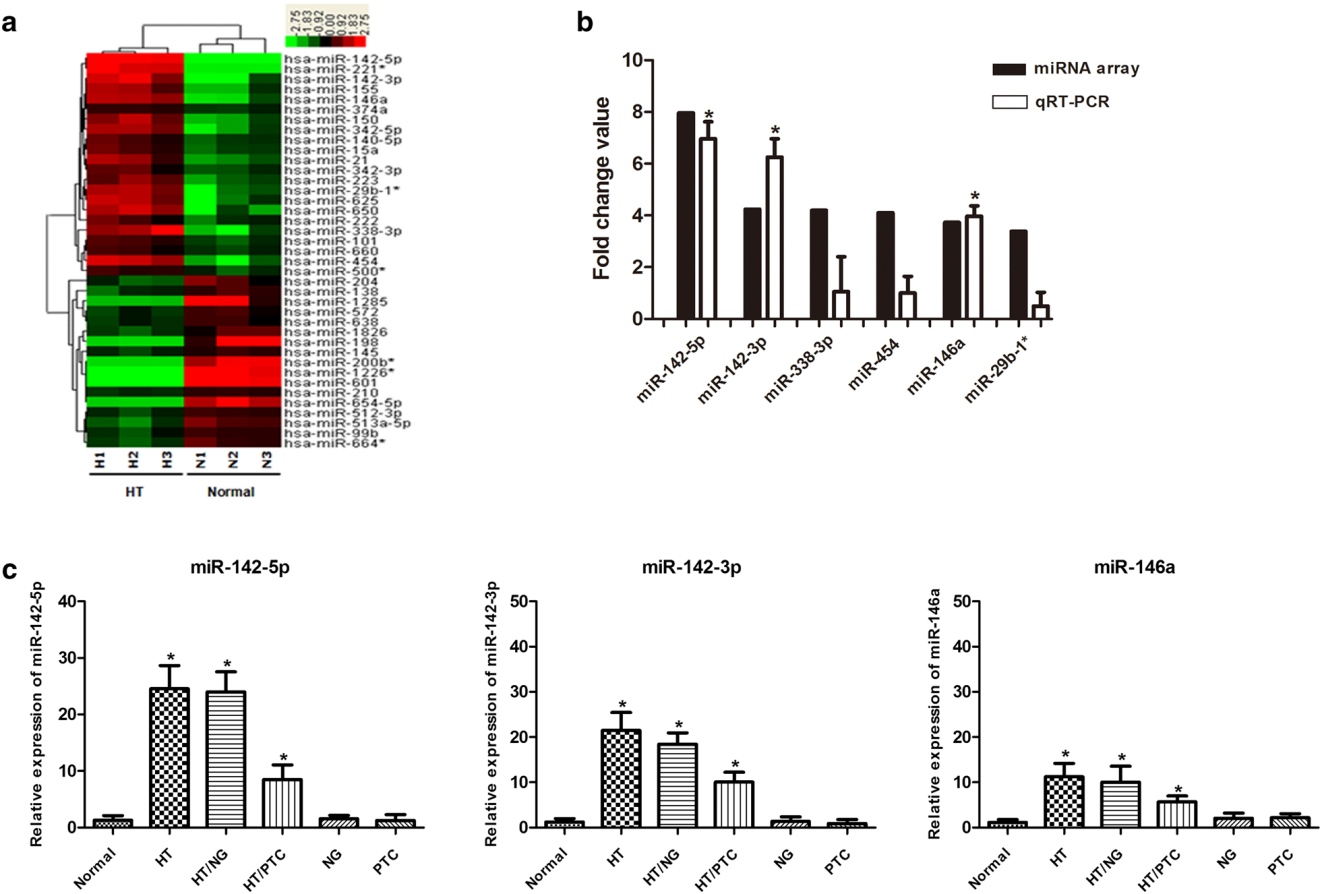
Dysregulated expression of miRNAs in HT. **a** Microarray data with significant gene expression changes of ≥twofold (*P* < 0.05). In total, 39 miRNAs showed different expression profiles between HT and normal thyroid using miRNA microarray. Of these, 22 miRNAs were upregulated and 17 miRNAs were downregulated. **b** Comparison of miRNA fold changes using miRNA microarray and qRT-PCR analysis. The fold change value of each miRNA was calculated by comparing the expression levels of HT samples with normal thyroid (n = 10 for each group). A total of six miRNAs were selected and three of them were confirmed by qRT-PCR. *P < 0.001. **c** HT-associated upregulation of miR-142-5p, miR-142-3p, and miR-146a expression patterns in various types of thyroid disease. Normal thyroid tissues (n = 21); *HT* primary Hashimoto’s thyroiditis (n = 42); *HT/NG* Hashimoto’s thyroiditis with nodular goiter (n = 15); *HT/PTC* Hashimoto’s thyroiditis with papillary thyroid carcinoma (n = 14); *NG* nodular goiter (n = 30); *PTC* papillary thyroid carcinoma (n = 20). *P < 0.001

To identify miRNA expression levels in the HT thyroid gland, we performed miRNA microarray analysis using RNA obtained from three primary HT cases, three HT component of HT associated with PTC cases, and three normal thyroid cases. Of the 875 human miRNAs on the array (Sanger miRBase release 12.0), 39 miRNAs showed differential expression between HT and normal thyroid (Fig. 1a). In particular, **14** miRNAs were upregulated and **7** were downregulated more than twofold in HT vs. normal thyroid (Table 1). Of these, miR-142-5p displayed the most significantly upregulation, with a log2-fold change of 7.959 and *P* = 0.005. Comparing the primary HT and HT component of HT cases with PTC groups (Table 1), many of the same miRNAs were upregulated, including miR-142-3p, miR-338-3p, miR-454, miR-146a, miR-29b-1*, miR-150, and miR-223, or downregulated, including miR-654-5p, miR-601, miR-198, and miR-1226* (log2 FC ≥ 2, *P* < 0.05).

**Table 1:** Dysregulated expression microRNAs (P < 0.05) with at least twofold of log2 fold change obtained from comparisons in specimens from case and control participants respectively

HT group (HT vs. normal)			HT^a^ group (HT^a^ vs. normal)		
MicroRNA name	Log2 FC	P value	MicroRNA name	Log2 FC	P value
hsa-miR-142-5p	7.959	0.005	hsa-miR-338-3p	4.386	0.019
hsa-miR-221^a^	5.534	0.002	hsa-miR-29b-1^a^	4.083	0.012
hsa-miR-142-3p	4.236	0.011	hsa-miR-363	3.964	0.026
hsa-miR-338-3p	4.196	0.023	hsa-miR-454	3.946	0.021
hsa-miR-454	4.105	0.024	hsa-miR-194	3.71	0.031
hsa-miR-146a	3.731	0.016	hsa-miR-142-3p	2.698	0.036
hsa-miR-650	3.677	0.028	hsa-miR-342-5p	2.483	0.034
hsa-miR-29b-1^a^	3.39	0.026	hsa-miR-146a	2.406	0.029
hsa-miR-625	3.325	0.041	hsa-miR-150	2.211	0.021
hsa-miR-342-5p	3.278	0.020	hsa-miR-223	2.151	0.016
hsa-miR-150	3.111	0.006	hsa-miR-654-5p	−3.362	0.037
hsa-miR-155	3.067	0.011	hsa-miR-601	−5.076	0.011
hsa-miR-21	2.931	0.007	hsa-miR-198	−5.536	0.038
hsa-miR-223	2.446	0.007	hsa-miR-1226^a^	−6.017	0.001
hsa-miR-513a-5p	−2.057	0.009			
hsa-miR-1285	−4.761	0.037			
hsa-miR-654-5p	−4.861	0.005			
hsa-miR-200b^a^	−5.122	0.004			
hsa-miR-198	−5.514	0.038			
hsa-miR-601	−5.957	0.001			
hsa-miR-1226^a^	−5.996	0.001			

*HT* primary Hashimoto’s thyroiditis

*HT* ^a^ HT component of HT cases with papillary thyroid carcinoma

### Microarray validation of miRNAs differentially expressed in HT and normal thyroid tissues by qRT-PCR

To validate the miRNA microarray data, we performed qRT-PCR to analyze the expression level of miR-142-5p, which was the highest expression miRNA in pure HT, and the top upregulated miRNAs overexpressed both in pure HT tissues and HT tissues adjacent to PTC, including miR-142-3p, miR-146a, miR-338-3p, miR-29b-1*, and miR-454 in ten HT samples. The fold change was calculated by comparing the relative expression level of each miRNA in the HT and normal groups. Three selected miRNAs (miR-142-5p, miR-142-3p, and miR-146a) were consistently confirmed to be highly expressed in HT tissues (Fig. 1b; *P* < 0.001). Next, we investigated the specific miRNA expression in various types of thyroid disease and found that the expression levels of miR-142-5p, miR-142-3p, and miR-146a were significantly higher in HT with or without other diseases than in nodular goiter or primary PTC (Fig. 1c, *P* < 0.001).

### Specific miRNA expression in HT associated with thyroglobulin antibody (TgAb)

**Fig. 2:**
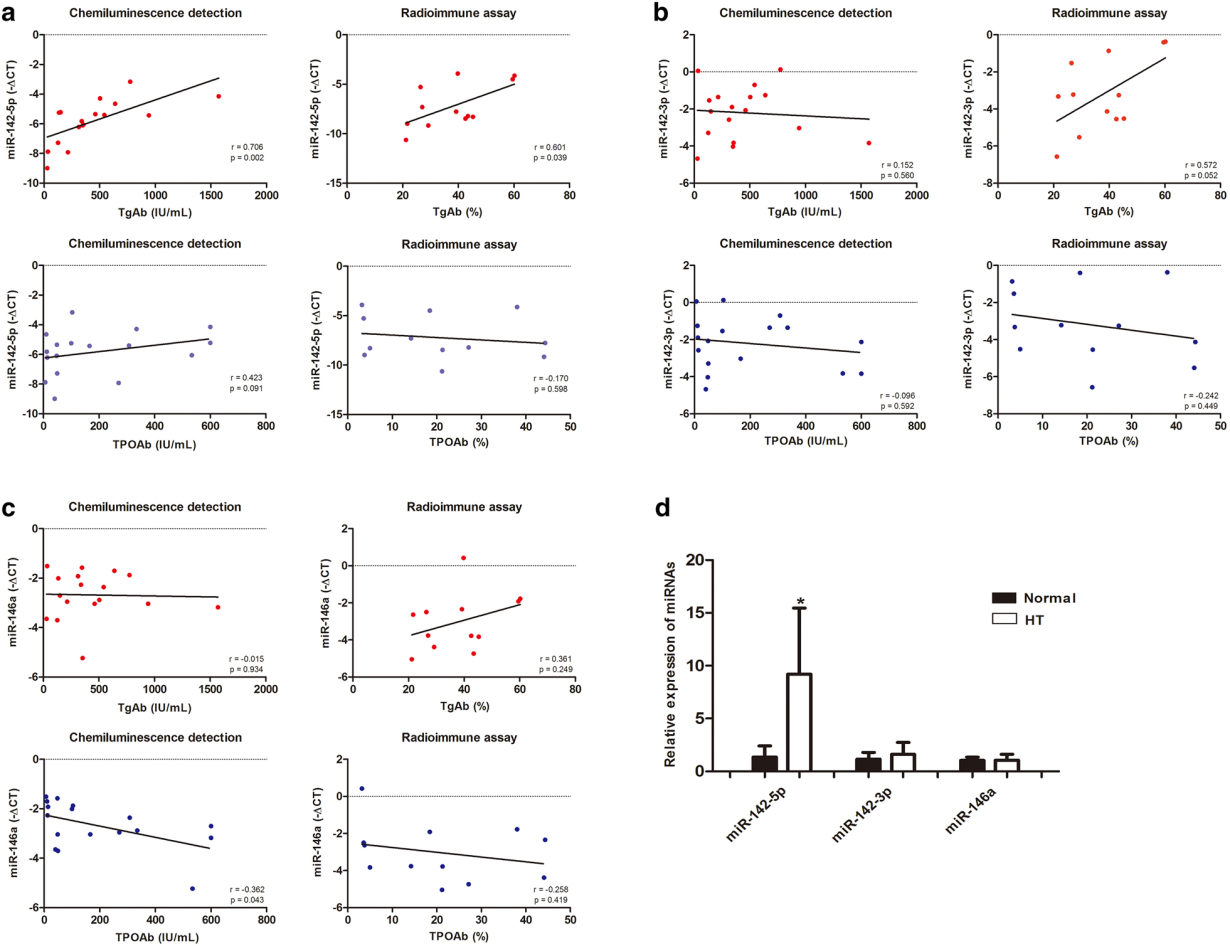
Relationship between miRNA overexpression and clinical data. **a** The relationship between the expression of miR-142-5p in thyroid tissue and the level of TgAb or TPOAb in serum using chemiluminescence detection (*left* n = 17) or using radioimmune assay (*right* n = 12). **b** The relationship between the expression of miR-142-3p in thyroid tissue and the level of TgAb or TPOAb in serum using chemiluminescence detection (*left* n = 17) or using radioimmune assay (*right* n = 12). **c** The relationship between the expression of miR-146a in thyroid tissue and the level of TgAb or TPOAb in serum using chemiluminescence detection (*left* n = 17) or using radioimmune assay (*right* n = 12). **d** Detection of the dysregulated expression miRNA in serum of HT patients (n = 5) and healthy donors (n = 6).*P = 0.032

As the progress and diagnosis of HT depends mainly on detecting abnormal levels of serum markers, especially thyroid autoantibodies, we investigated the association of miR-142-5p, miR-142-3p, and miR-146a expression with clinical data. We found that only miR-142-5p overexpression was positively associated with increased TgAbs in serum of HT patients using chemiluminescence detection and radioimmune assay (Fig. 2a–c) (r = 0.706, *P* = 0.002, and r = 0.60, *P* = 0.039 respectively) but miR-142-5p expression did not correlate with the level of thyroid peroxidase antibody (TPOAb), thyroid-stimulating hormone (TSH), or Thyroglobulin (Tg).

We also compared serum expression levels of miR-142-5p, miR-142-3p, and miR-146a between patients with HT and healthy controls and found that miR-142-5p significantly increased in the serum of HT patients (Fig. 2d). By contrast, the expression of miR-142-3p and miR-146a were no different between HT patients and healthy controls.

### Detection of miR-142-5p expression pattern by ISH using LNA-modified oligonucleotide probes

**Fig. 3:**
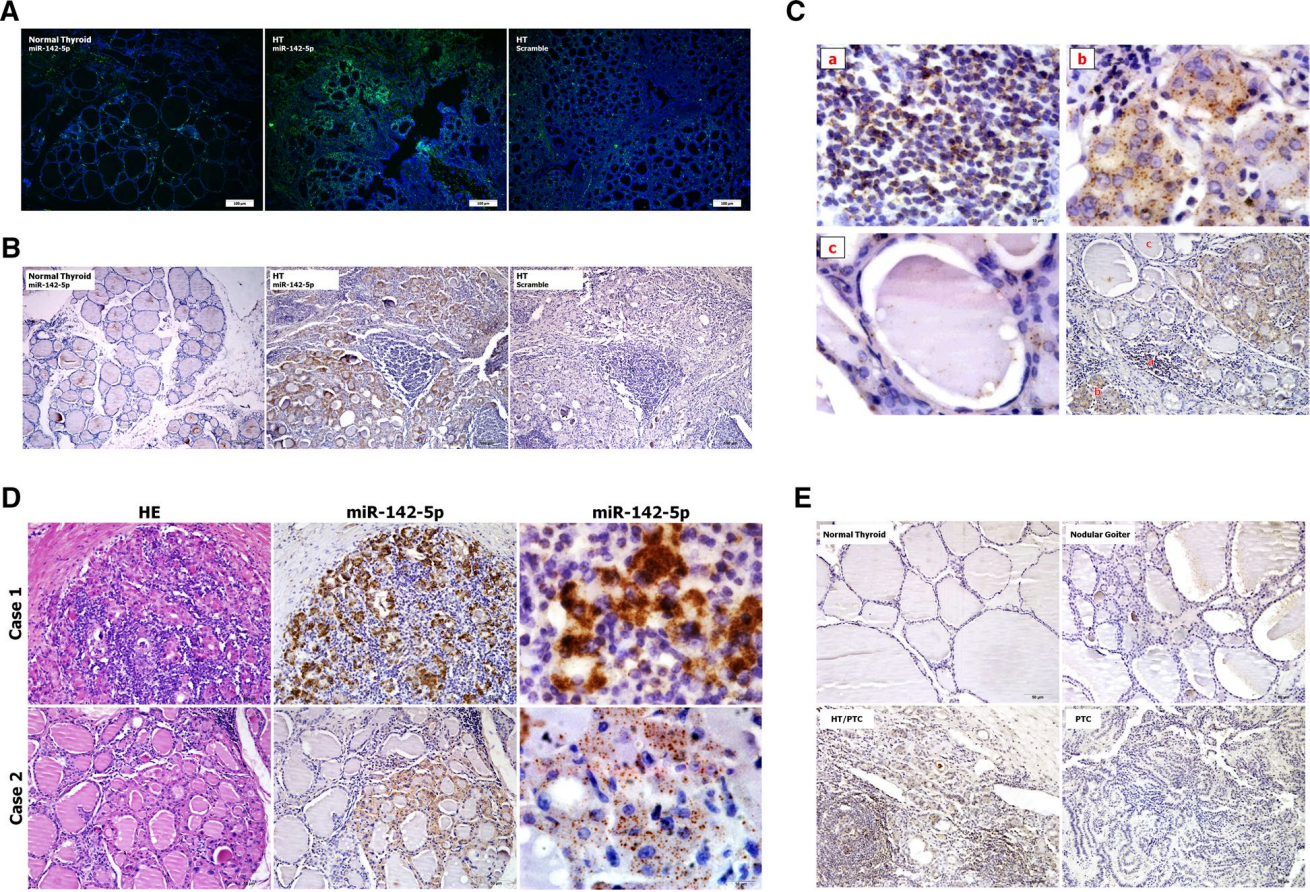
LNA-ISH of miR-142-5p in FFPE samples. **A** 5ʹ-FITC labeling LNA-ISH for miR-142-5p detection (×100). Green staining of miR-142-5p was visible in part of follicular epithelium. **B**–**E** 3ʹ,5ʹ-digoxin double labeling LNA-ISH for miR-142-5p detection. **B** Differential expression of miR-142-5p between normal thyroid and HT (×100). **C** The specific expression region of miR-142-5p in HT (*bottom right* ×200), including the ectopic lymphocyte (*top left a* ×1000), injured follicular epithelial cells (*top right* *b* ×1000) and normal follicular epithelium adjacent to the lesion (*bottom left c* ×1000). **D** Hematoxylin-eosin staining and LNA-ISH with serial section and the different intensity of miR-142-5p in HT (*left and middle* ×200, *right* ×1000). Case 1 showed mass-like positive signals and case 2 showed dot-like positive signals. **E** Expression patterns of miR-142-5p in various types of thyroid disease (×200). miR-142-5p is only positive in the HT with PTC cases

As LNA-modified oligonucleotide probes are sensitive for miRNA detection in FFPE samples [14], we first performed FITC-labeling LNA-ISH for miR-142-5p using the FFPE samples of HT thyroid tissues. The expression of miR-142-5p was detectable in HT samples but not in the normal thyroid (Fig. 3A). We also detected miR-142-5p by combining LNA-ISH with the biotin-free tyramide signal amplification system to observe the distribution in better tissue structure. Using this method, DAB staining of miR-142-5p was a visible brown color in the cytoplasm (Fig. 3B). Unexpectedly, overexpression of miR-142-5p was observed not only in lymphocytes (Fig. 3C, a) but also in the adjacent injured follicular epithelial cells (Fig. 3C, b). However, increased miR-142-5p expression was barely detectable in normal follicular epithelial cells in the same lesion (Fig. 3C, c). H&E staining and LNA-ISH of sequential sections further confirmed that miR-142-5p was overexpressed in eosinophilic follicular epithelial cells, which were the characteristic pathological change of HT (Fig. 3D, ×200). In addition to the most common expression pattern of punctate particle coloring in cytoplasm, a few samples showed dense mass-like coloring in the cytoplasm (Fig. 3D, ×1000) which was accompanied by severe fibrosis and morphologically damaged follicular epithelial cells. Meanwhile, overexpression of miR-142-5p was not observed in the normal thyroid, nodular goiter, or primary PTC, but could be seen in the HT with PTC cases (Fig. 3E).

### Identification of miR-142-5p target mRNA in thyrocytes

To identify the target gene of miR-142-5p, we infected primary thyrocytes with either mir-142 expression lentivirus vector (LV-has-mir-142) or those with control vector (LV-EGFP). At 72 h after infection, the infection efficiency was evaluated by visualization of EGFP using a fluorescent microscope (Additional file 1: Figure S1a). The expression of transducted miRNAs was confirmed using qRT-PCR assays which showed that miR-142-5p and miR-142-3p transcripts were increased 8.3- and 9.2-fold respectively (Additional file 1: Figure S1b).

**Fig. 4:**
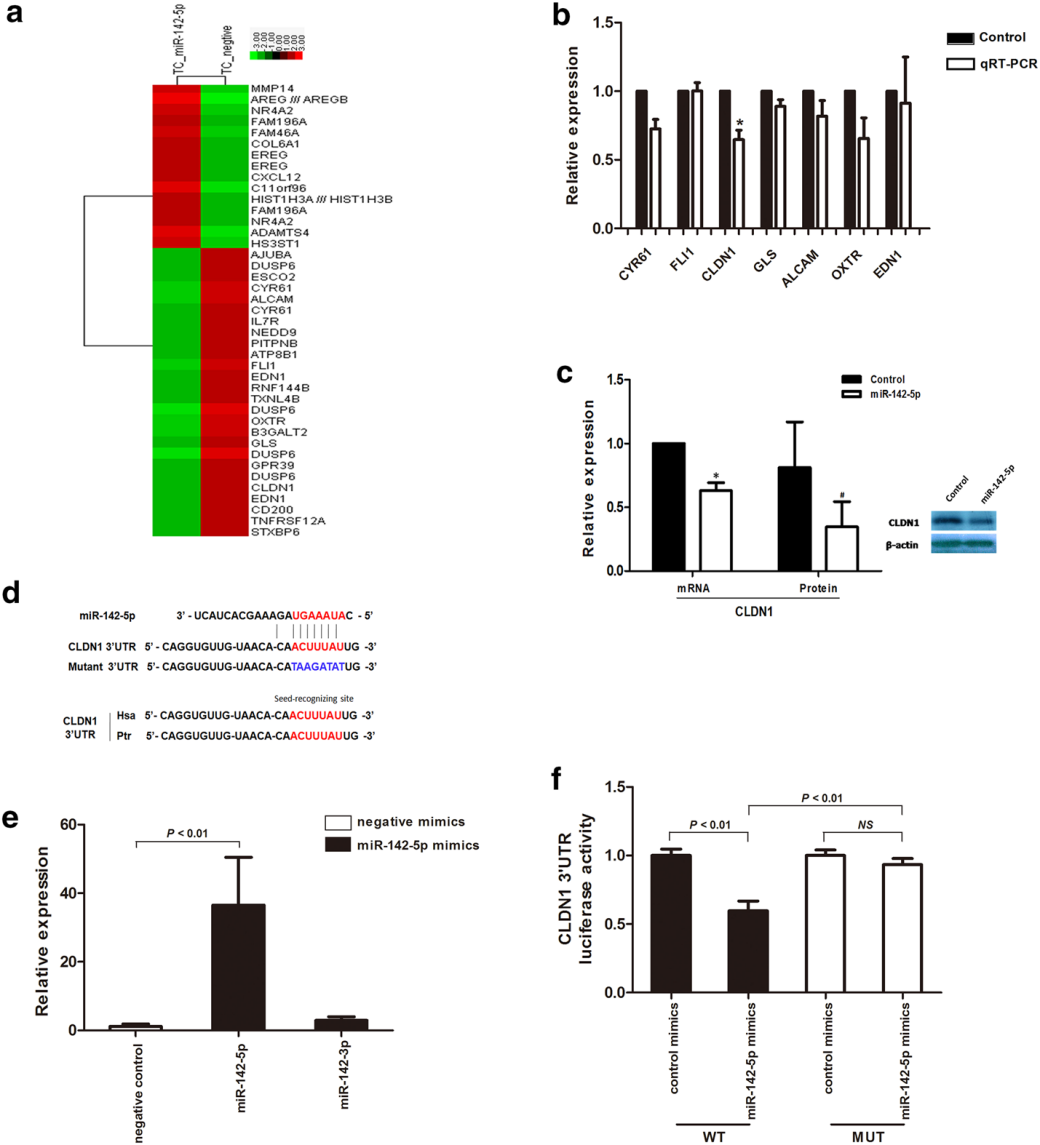
miR-142-5p inhibits CLDN1 expression in vitro. **a** The differential gene expression by gene array comparing the primary thyrocytes upregulated miR-142-5p with lentivirus vector and control vector. **b** The potential gene candidates were investigated using qRT-PCR in miR-142-5p transducted thyrocytes, the level of CLDN1 expression showed most reduction after transduction. *P = 0.032. **c** The expression of CLDN1 mRNA measured using qRT-PCR and protein by Western blot was analyzed after infection with miR-142 expression lentivirus or negative control in thyrocytes. *P = 0.032, ^#^ *P* = 0.009. **d** Luciferase reporter constructs of wild-type and mutant target sites in the 3ʹ-UTR of CLDN1 mRNA.* Red* indicates the miR-142-5p binding site, which was conservative in Homo sapiens (Hsa) and Pan troglodytes (Ptr). *Blue* indicates mutant sequences. **e** The transfection of miR-142-5p mimics dramatically increased the expression of miR-142-5p but not of miR-142-3p in HEK293T. **f** Luciferase construct containing the miR-142-5p binding site in the CLDN1 3ʹ-UTR (wild-type or mutant) was transfected and assayed in HEK293T. *NS* not significant

TargetScan 6.2 was used to predicted the targets of miR-142-5p,and the gene expression profile was used to identify potent candidates by comparing the primary thyrocytes, which had upregulated miR-142-5p by LV-has-mir-142 vector, with the one infected by the control vector. Among the potential targets (Fig. 4a), we focused on CLDN1 because it was downregulated in the thyrocytes after infection with LV-has-mir-142 using qRT-PCR (Fig. 4b) and was lost in various autoimmune diseases, including autoimmune thyroid disease [16, 17]. Comparing CLDN1 transcript and protein levels in thyrocytes transduced with LV-has-mir-142 vector and those with control vectors using qRT-PCR and Western blotting, we observed a clear reduction in the steady-state levels of CLDN1 by exogenous miR-142-5p expression (Fig. 4c).

To confirm that CLDN1 is a direct target of miR-142-5p, we generated a firefly luciferase reporter plasmid fused downstream to a segment of the CLDN1 3ʹ-UTR containing either the wild-type putative miR-142-5p binding sequence or the mutation sequence (Fig. 4d). After ensuring the mimics of miR-142-5p only induced miR-142-5p overexpression (Fig. 4e), the constructs were then cotransfected into HEK293T cells with miR-142-5p mimics or control mimics and luciferase activity was measured 48 h later. miR-142-5p significantly reduced CLDN1 WT-luciferase activity (*P* < 0.01) but failed to inhibit CLDN1 Mut-luciferase activity (Fig. 4f). Taken together, these data strongly suggest that miR-142-5p upregulation contributes to the reduction of CLDN1 protein levels.

**Fig. 5:**
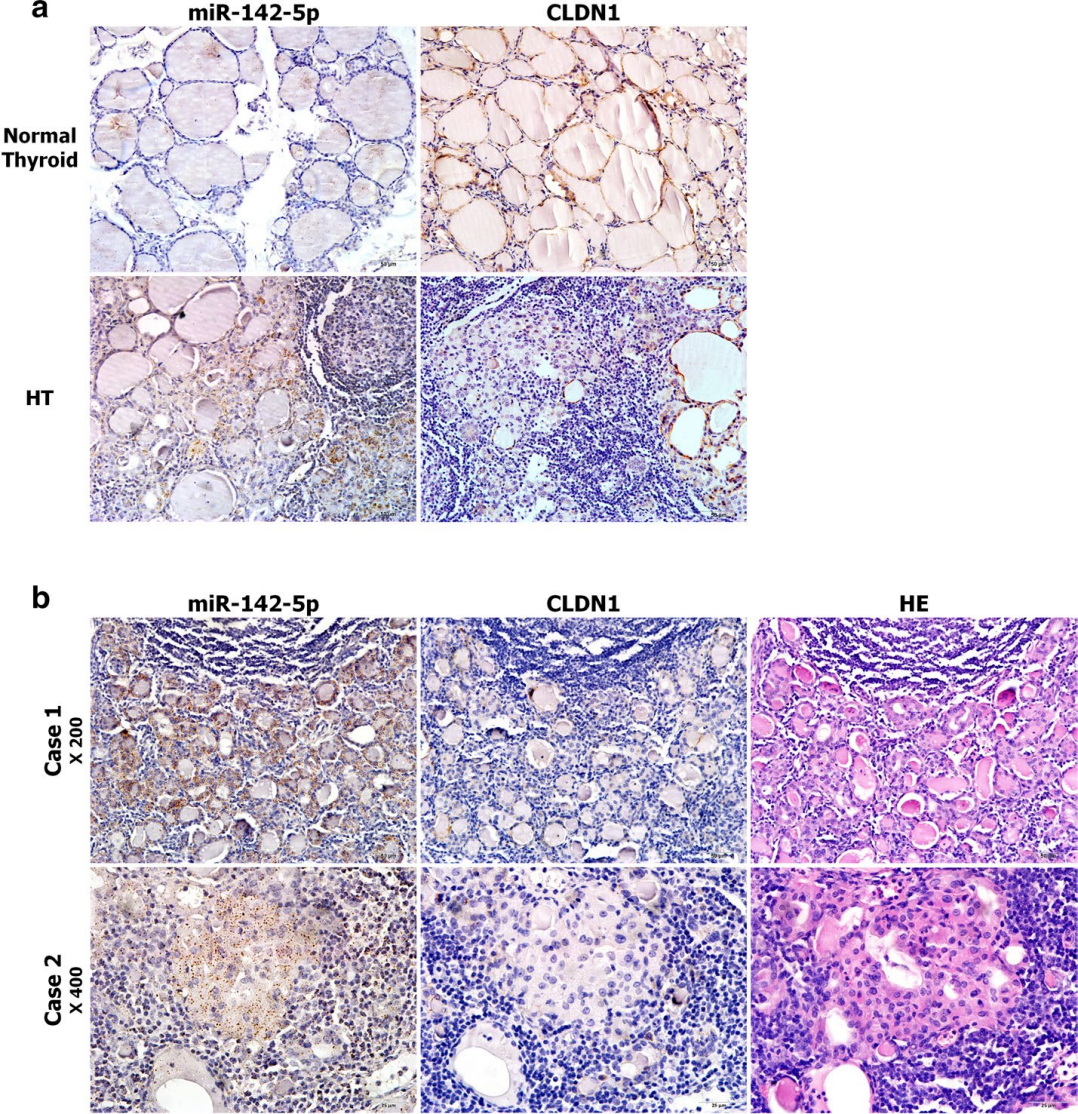
Immunostaining of claudin-1 (CLDN1) compared with the ISH of miR-142-5p in FFPE samples. **a** The mutually exclusive patterns between miR-142-5p (*left*) and CLDN1 (*right*) expression in normal thyroid and HT tissues (×200). **b** Images from serial FFPE HT tissue sections in which LNA-ISH for miR-142-5p (*left*), immunostaining for CLDN1 (*middle*) and HE staining (*right*) were done (*top* ×200, *below* ×400)

We next performed immumohistochemical staining of CLDN1 in FFPE samples and found that CLDN1 expression in normal thyroid follicular cells was missing miR-142-5p expression. Even in the same slide of HT, the expression of CLDN1 was high in the normal thyroid follicle but non-detectable in eosinophilic epithelial cells, which was opposite to the expression of miR-142-5p (Fig. 5a). As shown in the images of serial sections of HT (Fig. 5b), lack of CLDN1 expression was seen in the areas with abundant miR-142-5p expression.

### miR-142-5p impaired the human thyroid epithelial barrier function by negative regulating CLDN1

**Fig. 6:**
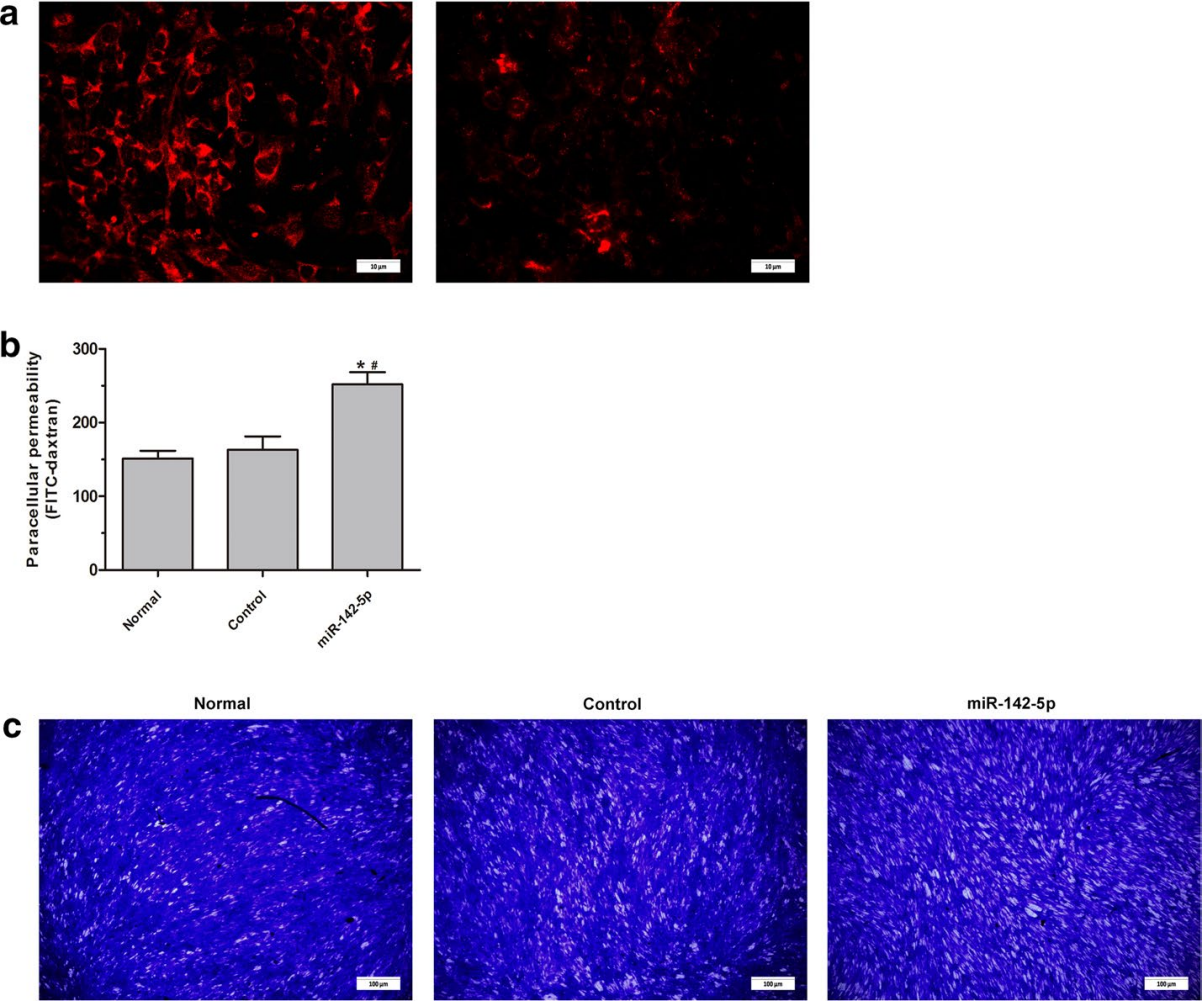
Immunofluoresence staining and permeability test of thyrocytes. **a** Immunofluoresence staining of CLDN1 in thyrocytes transfected with negative control lentivirus vector (linear distribution of CLDN1, *left* ×1000) and mir-142 lentivirus vector (discontinuous and diminished staining pattern, *right*, ×1000). **b** Permeability test of the thyrocyte monolayer, which showed the increased permeability of the monolayer thyrocytes (*P < 0.01 vs. normal, ^#^ *P* < 0.01 vs. control). **c** Cells staining of the thyrocyte monolayer after permeability testing, which showed the increased of the intercellular gap (×100)

As CLDN1 is an essential molecule in tight junctions and was found to be lost in pathological thyroid epithelial cells in HT (Fig. 5), we performed immunofluorescence staining on confluent thyrocytes with or without transduction of miR-142-5p. Unlike the linear distribution of CLDN1 along the entire cell–cell contact in normal and control thyrocytes, upregulation of miR-142-5p obtained a discontinuous and diminished staining pattern (Fig. 6a).

To confirm the immunofluorescence results, permeability of the monolayer was assessed via the addition of a FITC-Dextran solution to the thyrocyte monolayer followed by fluorescence analysis. The result showed that relative fluorescence unit (RFU) in the upregulation of miR-142-5p was much higher than in the control (258.64 ± 14.44 vs. 166.75 ± 25.35 RFU, *P* < 0.01) or normal group (258.64 ± 14.44 vs. 148.32 ± 13.38, *P* < 0.01) (Fig. 6b, c).

## Discussion

With increasing recognition that dysregulated miRNA expression exists in many autoimmune diseases [18], disease-associated miRNAs have a crucial pathogenic role in the development of autoimmune diseases [19, 20]. Our results showed that 39 miRNAs showed dysregulated expression in HT (*P* < 0.05, fold change >2). Most of altered miRNAs, including miR-142, miR-155, miR-150, miR-223, miR-146, and miR-21, had been reported regulating immune responses and related to various autoimmune diseases [21–25]. This may be due to the thyroid gland being attacked by various cell- and antibody-mediated immune processes and invasion of the leukocytes. These data could predict the miRNA expression profile of HT which is differentiated from normal thyroid and provide theoretical basis for the further study of functional miRNA in HT. We subsequently verified the miRNA candidates with enlarged samples and found that miR-142-5p, miR-142-3p, and miR-146a were overexpressed in HT, which is consistent with the results of microarray analysis. The same findings were observed by comparing nodular goiter and PTC with or without HT. These results suggest that miR-142-5p, miR-142-3p, and miR-146a were closely associated with HT.

The diagnosis of HT is made mainly by detecting elevated levels of thyroid autoantibodies in the serum [26]. We found that the level of TgAb was positively only correlated with the expression of miR-142-5p, although TPOAb is the most common anti-thyroid autoantibody present in approx. 90 % of HT [27, 28]. This might be due to the different distribution of Tg and TPO in the thyroid follicular epithelium. Tg is secreted by thyroid follicular epithelial cells and stored in the thyroid follicular cavity, which has strong immunogenicity [29]. Whereas, TPO is located at the apical surface of the thyroid follicular epithelial cells, which may move to the basal side through lateral diffusion of the membrane and be recognized by macrophages or dendritic cells in the intercellular space [30]. In thyroiditis, TgAbs precede the appearance of thyroperoxidase antibodies in mouse models of spontaneous autoimmune disease [31]. Thus miR-142-5p may participate in the early reaction of the autoimmune response in HT.

Circulating miRNAs, as a novel class of potential biomarkers for diagnosis or prognosis, have recently received much attention not only in cancer but also in autoimmune diseases [32–34]. In this retrospective study, although we have found that only the level of miR-142-5p was detectable in patients with HT vs. healthy controls in serum samples, for verifying miR-142-5p expression in the serum of HT patients, further studies on large serum sample size need to be performed in a prospective study. Studies have indicated that circulating miRNAs are ideal biomarkers for cancer detection should be tissue specific and frequently dysregulated in cancer, in addition to their stability in the circulation [35]. Thus, together with the findings that miR-142-5p was the highest expression miRNA in thyroid tissues of HT and relevant to the level of TgAb, miR-142-5p is likely to be a potential therapeutic target of HT. The LNA-modified oligonucleotide is one of the most sensitive probes currently available for miRNA detection, even in FFPE tissues [14, 15]. We applied this method to LNA-ISH and were able to sensitively detect miR-142-5p expression in HT thyroid tissues but not in the normal thyroid, which was consistent with the qRT-PCR results. miR-142-5p together with miR-142-3p, is known to be two transcripts of the has-miR-142 locus [36] which has been reported to be hematopoiesis-specific miRNAs [37, 38]. Thus studies have focused on its dysregulated expression and immune regulating function in peripheral blood mononuclear cells of autoimmune diseases [21, 39]. However, recent studies have showed that miR-142-5p was expressed in hippocampal neurons [40] and proliferative vascular smooth muscle cells [41]. Intriguingly, we found that the overexpression of miR-142-5p was observed not only in lymphocytes but also in HT injured thyroid epithelial cells, which suggests that miR-142-5p may have a crucial role in the characteristic lesion of HT.

Furuse et al. first cloned *CLDN1* from chicken liver and characterized its key role in the tight junctions [42]. It is very important number of CLDN family proteins which are the most important components of the tight junctions [43, 44]. More evidence has shown that CLDN1 expression is downregulated in various autoimmune diseases [17, 45–48]. In the present study we found that CLDN1 mRNA and protein expression are downregulated by miR-142-5p. Using the luciferase assay we showed that miR-142-5p can bind directly to the CLDN1 3′-UTR in HEK293T. Thus the results suggest that *CLDN1* is the target gene of miR-142-5p in vitro. In addition, CLDN1 encoded by the *CLDN1* gene was highly expressed in normal thyroid epithelium, but reduced in HT injured thyroid epithelial cells. Our ISH analyses further indicated that cells with reduced CLDN1 expression frequently had increased miR-142-5p expression, which was non-detectable in normal thyroid epithelium. These mutually exclusive expression patterns support the hypothesis that *CLDN1* is a potent target gene of miR-142-5p in vivo.

The functional unit of the thyroid is the follicle composed of single-layered epithelium and the follicular lumen, in which Tg is stored and iodothyronines are synthesized. The junctional complex of thyroid follicular cells consists of tight junctions and adherens junctions, which limit paracellular permeability. CLDN1 is a membrane protein that belongs to the claudin family and a component of tight junctions serving as a physical barrier to prevent solutes from passing freely through the paracellular space. Studies have shown that downregulated CLDN1 contributes to autoimmune diseases [17, 45–48] and particularly impairs the epithelial barrier function in primary cultured human thyrocytes [16, 30]. Similarly, we found that artificially raised expression of miR-142-5p in thyrocytes obtained a diminished staining pattern of CLDN1 and increased the permeability of the monolayer thyrocytes. Thyrocytes hold a unique position similar to the blood–brain barrier, making a tight barrier between the extracellular compartments, the lumen, and the extra follicular space. We hypothesized that miR-142-5p can break the paracellular barrier by downregulating CLDN1, which allows the exposure of normally secluded autoantigens, Tg, and TPO to the immune system. Thus it might contribute to the occurrence of HT.

## Conclusions

We, for the first time, systematically screened the miRNA expression profile of HT thyroid gland and found multiple miRNAs dysregulated expression, of which miR-142-5p, miR-142-3p, and miR-146a are confirmed to be overexpressed. Clinical data showed that only miR-142-5p is positively correlated with TgAb and can be detected in patient serum. Interestingly, the expression of miR-142-5p was also found in characteristic Hürthle cells and *CLND1* was demonstrated to be the direct target gene of miR-142-5p, suggesting that miR-142-5p may impair the human thyroid epithelial barrier function by downregulating CLDN1 expression to participate in the pathologic changes of HT. Taken together, our results implied that miR-142-5p may contribute to the pathogenesis of HT and may be considered as a potential molecular target in future studies.

## Additional file

10.1186/s12967-016-0917-6 Clinical characteristics of the 142 patients. **Figure S1.** Generation of lentiviral vectors and miRNA transfer.

## Abbreviations

RT-PCR: real time-polymerase chain reaction
CLDN1: claudin-1
HT: Hashimoto’s thyroiditis
UTRs: untranslated regions
FFPE: formalin-fixed paraffin-embedded
PTC: papillary thyroid carcinoma
LNA: locked nucleic acid
ISH: in situ hybridization
PBS: phosphate buffered saline
FITC: fluorescein isothiocyanate
BSA: bovine serum albumin
SSC: saline sodium citrate
TBST: tris buffered saline with tween
DAB: diaminobenzidine
TgAb: thyroglobulin antibody
TPOAb: peroxidase antibody
TSH: thyroid-stimulating hormone
Tg: thyroglobulin
LV: lentivirus vector
EGFP: enhanced green fluorescent protein
RFU: relative fluorescence unit
