# Supplementary material for: MicroRNA-142-5p contributes to Hashimoto’s thyroiditis by targeting CLDN1
Source: J Transl Med. 2016 Jun 8;14:166. doi: 10.1186/s12967-016-0917-6 (PMC4898455; doi:10.1186/s12967-016-0917-6)
Supplement: Supplementary file 1 — 10.1186/s12967-016-0917-6 Clinical characteristics of the 142 patients. Figure S1. Generation of lentiviral vectors and miRNA transfer. [file 12967_2016_917_MOESM1_ESM.doc]

**Table S1.** Clinical characteristics of the 142 patients.

|  |  |  | **HT** |  |  |  |
| --- | --- | --- | --- | --- | --- | --- |
|  | **Normal** | **primary HT** | **HT/PTC** | **HT/NG** | **PTC** | **NG** |
| ***n* (female/male)** | 21(15/6) | 42(39/3) | 14(13/1) | 15(13/2) | 20(14/6) | 30(23/7) |
| **Age** | 37.0 ± 14.6 | 54.1 ± 9.27 | 36.0 ± 14.18 | 34·5 ± 14·7 | 39.9 ± 11.53 | 33·4 ± 14·3 |
| **Family history** | None | None | None | None | None | None |
| **ophthalmopathy** | None | None | None | None | None | None |

HT: Hashimoto’s thyroiditis.

HT/PTC: Hashimoto’s thyroiditis concomitant thyroid carcinoma.

HT/NG: Hashimoto’s thyroiditis concomitant nodular goiter.

NG: nodular goiter.

**Figure S1.**


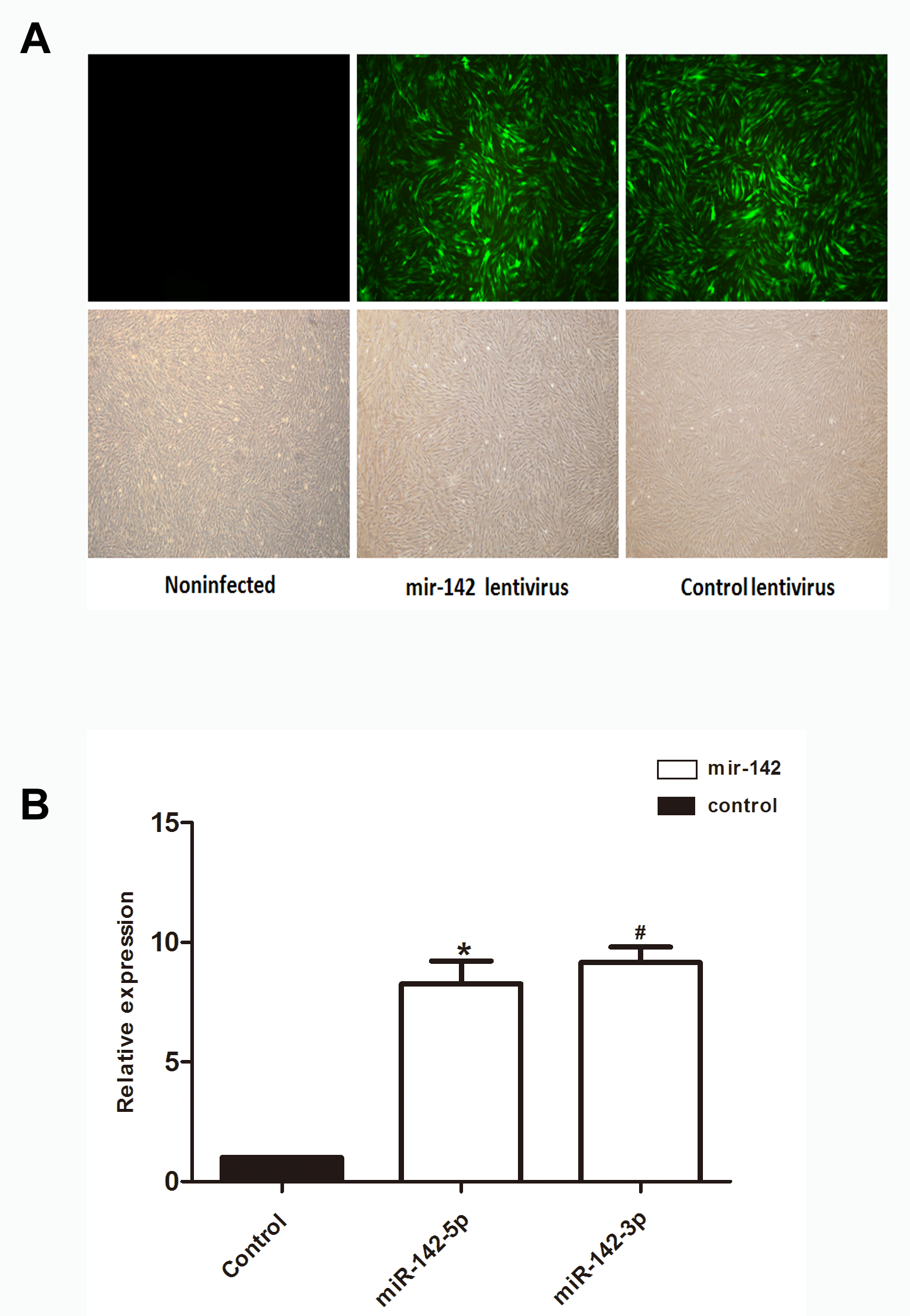


**Figure S1.** Generation of lentiviral vectors and miRNA transfer. (A) The infection efficiency of lentivirus in primary thyrocytes was evaluated by the expression of EGFP in cells (×100). (B) The level of miR-142-5p was determined in thyrocytes infected with Lv-mir-142 or control by qRT-PCR. **P*=0.01, #*P*<0.01.
